# Evaluation of vascular features of vocal cords proposed by the European Laryngological Society

**DOI:** 10.1007/s00405-017-4791-5

**Published:** 2017-10-30

**Authors:** Robert Šifrer, Johannes A. Rijken, C. René Leemans, Simone E. J. Eerenstein, Stijn van Weert, Jan-Jaap Hendrickx, Elisabeth Bloemena, Derrek A. Heuveling, Rico N. P. M. Rinkel

**Affiliations:** Department of Otolaryngology-Head and Neck surgery, VU University Medical Center, De Boelelaan 1117, 1081 HV Amsterdam, The Netherlands

**Keywords:** Glottis, Cancer, Narrow-band imaging, Longitudinal vascular patterns, Perpendicular vascular patterns

## Abstract

A newly proposed classification by the European Laryngological Society (ELS) of glottic lesions by narrow-band imaging (NBI) divides their vascular patterns into longitudinal and perpendicular ones. The latter are further subdivided into the wide and narrow patterns. The longitudinal, wide, and narrow patterns are characteristic of benign disease, papilloma, and malignancy, respectively. The aim of the study was to investigate the diagnostic effectiveness of the classification. Forty patients with glottic lesions underwent microlaryngoscopy. The vascular patterns of all vocal cords were defined with NBI. The affected vocal cords were histologically analysed and comprised the arm (A). Unaffected vocal cords were not histologically analysed but followed-up and comprised the arm (B) and were regarded as true negatives if no suspicious changes appeared during the follow-up. The vocal cords from the arm A were categorised into the benign and malignant group according to the histologic result. The ratio of vascular patterns was determined and the groups were statistically compared using the Chi-square test and Fisher’s exact test. Perpendicular changes were observed in 36.6% (9/26) of benign diseases and in 100% (23/23) of cancer conditions (*p* < 0.001). Wide perpendicular changes appeared only in papillomas (6/6) while narrow ones mostly in malignancies (23/26) and also in benign conditions (3/26) (*p* < 0.001). The sensitivity, specificity, positive and negative predictive values, and accuracy were 100, 95, 88, 100 and 96%, respectively. The new ELS classification can be used effectively and safely to differentiate malignant from benign disease.

## Introduction

Narrow-band imaging (NBI) is a reliable diagnostic tool that is in use for the early detection of head-and-neck cancer. Its application of narrow-band spectrum optical filters enhances the visualisation of mucosal and sub-mucosal microvascular structures [1, 2]. NBI is beneficial due to exploiting the fact that tumours need the recruitment of the surrounding blood vessels to support their growth and metabolic requirements [3]. The mucosal microvascular proliferation and changes are one of the first signs of malignant alteration and are associated with carcinogenesis [4, 5]. Thus, the key advantage of NBI endoscopy is the earlier recognition of oncologically suspicious mucosal lesions in comparison to the standard examinations. NBI-positive signs predict histological high-grade epithelium/dysplasia [6] and carcinoma reliably with a sensitivity (SE), specificity (SP), positive predictive value (PPV), negative predictive value (NPV), and accuracy (ACC) of 98, 90, 86, 88, and 92%, respectively [7].

NBI-positive signs are traditionally considered as well-demarcated brownish areas or scattered, irregular, thick, brown spots of different shapes and sizes and an afferent hypertrophic vessel that branches out in small vascular loops in the context of the lesion [7, 8]. To take a full advantage of NBI endoscopy of mucosal laryngeal lesions, a useful and simple classification is prerequisite.

The most commonly used classification of laryngeal glottic lesions by NBI is the one proposed by Ni and has been in use since 2011. It consists of five types, of which types I–IV are considered benign and type V malignant. The latter is further subdivided into type Va, Vb, and Vc. According to Ni, type Va may contain severe dysplasia and carcinoma in situ, while type Vb and Vc represent invasive cancer. It showed good diagnostic effectiveness for carcinoma with SE, SP, PPV, and NPV of 88.9, 93.2, 90.9, and 91.7%, respectively [9, 10]. Similar results were recently obtained by other authors using Ni classification [3, 8].

Although the Ni classification is regarded reliable and is widely used, there are some flaws to address. First, lesions of type IV could not be considered uniquely benign. In our experience, they could also represent high-risk epithelium/dysplasia and malignancy as opposed to Ni. Second, the structure of the classification could be simplified. In 2015, the new classification for vascular changes in the lesions of vocal cords was proposed by the European Laryngological Society (ELS) [11] dividing vascular patterns dichotomously in two groups.

The longitudinal vascular patterns, such as ectasia, meander, increased number of vessels (i.e. increased density), and increased branches of vessels, remain only in two dimensions of the vocal cord (length and width) and are associated with benign conditions. Varicose and convolute are advanced vascular lesions representing manifestly deformed vessels. While convolutes remain within the vocal cord, varicoses project above its surface. Both are easily recognised and are still considered as longitudinal vessel changes.

In contrast to longitudinal patterns being spread only along the length and the width of the vocal cords, the perpendicular patterns project perpendicularly in the third dimension towards the surface of the epithelium—hence the name. They are endoscopically recognised as the so-called intraepithelial papillary capillary loops (IPCL). The angle of the turning point of the loops might be either wide or narrow corresponding with papilloma and malignancy, respectively. The aim of this study was to investigate the reliability and diagnostic effectiveness of the newly proposed classification by the ELS of vocal cords’ vascular patterns.

## Methods

The present study was conducted between October 2016 and April 2017 at the Department of Otolaryngology-Head and Neck Surgery, VU University Medical Center, Amsterdam, The Netherlands. This prospective study included 40 patients undergoing microlaryngoscopy under general anaesthesia for various lesions of the vocal cords. NBI of all vocal cords was performed, focusing on the vascular patterns according to the new classification of ELS. All procedures were performed by two simultaneously present experienced endoscopists (RNPMR, RŠ). NBI images were recorded and discussed by two above-mentioned authors and a final consensus assessment was made.

To perform NBI, we used a rigid video-endoscope of 0°, 30°, or 70° connected to an Evis Exera II CLV-180 light source, HDTV compatible Camera Head, and Evis Exera II CV-180 image processor (Olympus Medical Systems Corporation, Tokyo, Japan).

The vocal cords affected by the lesions were histologically analysed. They were included into the arm A of the study. The lesions were either excised by cold-steel instruments or by CO_2_ laser. In case of suspicion of malignancy, either a laser chordectomy with radical intention or a biopsy was performed if radiation was considered as superior option in terms of voice outcome. Specimens were assessed by a specialised head-and-neck pathologist (EB) who was blinded to the NBI characteristics of the lesions. Histopathological classification was done according to the dysplasia system of the WHO [12].

The unaffected contralateral vocal cords were included into the arm B. The vascular patterns of these vocal cords were defined with NBI as well, but the histological confirmation was not performed. It would be practically unfeasible and ethically unacceptable to do biopsies of normal vocal cords [7]. Nevertheless, none affected vocal cords were regarded as if they were histologically negative, i.e., true negative, if they showed no changes after 3 months at a repeated transnasal flexible or transoral rigid endoscopic examination in an outpatient setting. The principle that NBI “negative” areas did not receive histologic confirmation was already implemented by Piazza [7, 13].

The NBI-determined vascular patterns of vocal cords of the arm A were correlated to the histological results of the lesions and were subsequently categorised into two groups, i.e., those with benign disease and those with malignancy. The ratio of various vascular patterns was calculated and the groups were statistically compared. For comparative analyses, the Chi-square test or Fisher’s exact test were used.

SE, SP, PPV, NPV, and ACC were determined on the basis of both arms of the study. Statistical analyses were performed using IBMI SPSS Statistics version 22 (Chicago, IL).

## Results

A total of 40 patients (32 males, 8 females; mean age 63 years; range, 9–92) underwent endoscopic evaluation of both vocal cords with NBI during microlaryngoscopy. Of 80 vocal cords analysed in total, 49 affected vocal cords with NBI-determined vascular patterns and histological confirmation were categorised into the arm A. Thirty-one unaffected vocal cords with NBI-determined vascular patterns and no histologic analysis but follow-up were categorised into the study arm B.

Of 49 vocal cords from the arm A, the benign group included 26 vocal cords and the malignant group 23. Table 1 illustrates the relationship between endoscopic findings with NBI considering longitudinal and perpendicular microvascular patterns and final histological results in the arm A.

**Table 1:** Relationship between microvascular patterns defined by NBI and histological diagnosis in the study arm A, according to ELS classification [11]

Histological diagnosis (number of affected vocal folds)	Longitudinal (*N* = 49)	Perpendicular (*N* = 32)
Polyp [5]	5 (10.2%)	0 (0)
Cyst [1]	1 (2%)	0 (0)
Granuloma [1]	1 (2%)	1 (3.1%)
Inflammation [4]	4 (8.3%)	0 (0)
Hyperplasia [1]	1 (2%)	1 (3.1%)
Keratosis [1]	1 (2%)	0 (0)
Papilloma [6]	6 (12.2%)	6 (18.8%)
Mild dysplasia [3]	3 (6.1%)	0 (0)
Moderate dysplasia [4]	4 (8.3%)	1 (3.1%)
Severe dysplasia [5]	5 (10.2%)	5 (15.6%)
Carcinoma [18]	18 (36.7%)	18 (56.3%)

In the study arm A, longitudinal vascular patterns were identified in all vocal cords. Perpendicular changes, on the other hand, were observed in 36.6% (9/26) of benign diseases and in 100% (23/23) of cancer conditions (*p* < 0.001). There was no vocal cord cancer without perpendicular vascular patterns (Table 2).

**Table 2:** Distribution of perpendicular vascular changes in the benign and malignant group in the study arm A (Fisher exact test)

	Overall (*N* = 49)	Benign (*N* = 26)	Malignant (*N* = 23)	*p*
Perpendicular				< 0.001
Identified	32	9 (36.6%)	23 (100%)	
NOT identified	17	17 (65.4%)	0 (0%)	

According to ELS [11], we subdivided the perpendicular changes into the ones with narrow-angled (predicting carcinoma) and the ones with wide-angled turning points (predicting papilloma) (Table 3).

**Table 3:** Distribution of perpendicular vascular changes with wide- and narrow-angled turning points in the papillomas, benign, and malignant vocal cords in the study arm A (Chi-square test)

	Overall (*N* = 32)	Benign (*N* = 3)	Papilloma (*N* = 6)	Malignant (*N* = 23)	*p*
Perpendicular					< 0.001
Wide-angled	6	0	6	0	
Narrow-angled	26	3	0	23	

In 31 vocal cords comprising the study arm B, only longitudinal vascular patterns were identified with NBI. None showed signs of malignant alteration during the follow-up of 3–9 months. The longitudinal vascular patterns of these vocal cords were, therefore, considered true negative and were added to the amount of true negatives of the arm A. Altogether, we found 23 true positives, 54 true negatives, and 3 false positives. There were no false-negative results. Concluding from these data that included both study arm A and arm B the SE, SP, PPV, NPV, and ACC in our study were 100, 95, 88, 100, and 96% respectively.

## Discussion

In the present study, longitudinal vascular patterns were identified in all cases rendering them useless in predicting the correct histological diagnosis (Table 1).

The comparison of the benign and malignant groups in relation to the presence of perpendicular vascular changes (36.6 vs. 100%, *p* < 0.001, Table 2) supports the reliability of the new ELS classification in differentiating the benign lesions from malignant. In the light of these observations, assessment of perpendicular patterns seems to be *the first step* with NBI diagnostics. When absent it could be safely assumed that the lesion is benign. However, the presence of perpendicular features does not rule out benign disease, as in this analysis, 36.6% of benign lesions showed a perpendicular pattern.

Here, the subdivision of perpendicular vascular changes based on the shape of their turning points comes in useful. In fact, perpendicular changes are recognised by NBI as vessel loops arising from the depth of the vocal cord, projecting towards its surface, and then turning back into the depth. They have a tangled and spiralling course. As clearly described by Arens [11], the papilloma is associated with loops with wide-angled turning points embedded in a warty structure. In cancer, the turning points are narrow-angled and the loops are more spiralling. Distinguishing the two types of turning points is not always easy and experience is needed. All cases with wide-angled turning points were histologically papillomas, whereas 23 out of 26 cases of narrow-angled turning points were carcinomas. The remaining three cases were false-positive. This provides the evidence that *the second step* in the NBI diagnostics would be defining the type of perpendicular vascular pattern. This distinction could be safely used to differentiate the papilloma from carcinoma.

No current publication exists on histological verification of newly proposed guidelines of vascular changes in the vocal cords by ELS. Schossee discovered that significantly more changes can be detected by NBI in comparison to white light endoscopy with respect to longitudinal vessel changes [14].

The SE, SP, PPV, NPV, and ACC of 100, 95, 88, 100, and 96%, respectively, for NBI to predict malignancy of the vocal cords were registered in our study. Several reports in the literature have already addressed diagnostic effectiveness of NBI in this field. However, authors of the previous studies used Ni classification. Their results are as follows 88.9–97, 84.6–96, 90.9–93, 91.6–99, and 95.1–97%, respectively [3, 8–10]. The results of Piazza are very similar with values of 98, 90, 86, 88, and 92%, respectively, despite not using Ni classification. Instead, he used more traditional NBI-positive features [7].

Our results are comparable to the ones in the literature. Nevertheless, there are differences. It is evident that we achieved higher SE and NPV what is explained by no false-negative results. On the other hand, we obtained three false-positive cases. As a consequence, the PPV was lower in comparison to authors using Ni classification [3, 8, 9]. The definition of type IV lesion [9] is the main reason for these differences in outcome. Ni describes it as ICPL that are visible as relatively regularly arranged, scattered, small, dark brown spots of low density. Histologically, they correspond to mild-and-moderate dysplasia and no type IV patient had severely dysplastic or malignant lesion [9, 10]. In contrast, ELS classification regards the same lesion (relatively regularly arranged, scattered, small, dark brown spots of low density) as perpendicular, furthermore, perpendicular with narrow-angled turning points and, therefore, suspicious for cancer [11].

Moreover, Piazza describes NBI-positive signs in various publications as typical spots and dots that are scattered, thick, dark, brown, or brownish [2, 7, 13, 15–19]. These attributes correspond, both, to the description of perpendicular vascular change according to ELS classification and to type IV (and V) according to Ni classification. At this point, the classification of Ni regarding type IV lesion as benign and the classification of ELS and of Piazza regarding it as malignant differ considerably.

## Conclusions

Based on our results and the data from the literature, the newly proposed ELS classification of vascular changes of the vocal cords could effectively and safely be used to differentiate malignant from benign disease. Due to the dichotomous structure of the classification, it is simpler and seems easier to apply, making it more advantageous for a less experienced surgeon as opposed to Ni and the debatable type IV lesion definition. Taking into account the ELS classification, the endoscopy with NBI should be done in two steps: assessment of a possible perpendicular vascular pattern to differentiate between possible malignancy and benign disease. In the presence of a perpendicular pattern, step two will define narrow- and wide-angled turning points of the ICPL, differentiating between carcinoma and papilloma, respectively.
